# Nanofibrous Silver-Coated Polymeric Scaffolds with Tunable Electrical Properties

**DOI:** 10.3390/nano7030063

**Published:** 2017-03-13

**Authors:** Adnan Memic, Musab Aldhahri, Ali Tamayol, Pooria Mostafalu, Mohamed Shaaban Abdel-wahab, Mohamadmahdi Samandari, Kamyar Mollazadeh Moghaddam, Nasim Annabi, Sidi A. Bencherif, Ali Khademhosseini

**Affiliations:** 1 Center of Nanotechnology, King Abdulaziz University, Jeddah 21569, Saudi Arabia; musab.alzaheri@gmail.com (M.A.); mshabaan90@yahoo.com (M.S.A.); alik@rics.bwh.harvard.edu (A.K.); 2 Department of Biochemistry, King Abdulaziz University, Jeddah 21569, Saudi Arabia; 3 Biomaterials Innovation Research Center (BIRC), Department of Medicine, Brigham and Women’s Hospital, Harvard Medical School, Boston, MA 02139, USA; atamayol@mit.edu (A.T.); pooriam@mit.edu (P.M.); mehdisam@mit.edu (M.S.); Kamyar.Mollazadeh@gmail.com (K.M.M.); n.annabi@neu.edu (N.A.); 4 Harvard-MIT Division of Health Sciences and Technology, Massachusetts Institute of Technology, Cambridge, MA 02139, USA; 5 Wyss Institute for Biologically Inspired Engineering, Harvard University, Boston, MA 02115, USA; 6 Department of Chemical Engineering, Northeastern University, Boston, MA 02115-5000, USA; s.bencherif@northeastern.edu; 7 Sorbonne University, UTC CNRS UMR 7338, Biomechanics and Bioengineering (BMBI), University of Technology of Compiègne, BP 20529, Rue Personne de Roberval, 60205 Compiègne, France; 8 School of Engineering and Applied Sciences, Harvard University, Cambridge, MA 02138, USA; 9 Department of Bioindustrial Technologies, College of Animal Bioscience and Technology, Konkuk University, Seoul 05029, Korea

**Keywords:** electrospinning, electrical properties, nanocoatings, flexible electronics

## Abstract

Electrospun micro- and nanofibrous poly(glycerol sebacate)-poly(ε-caprolactone) (PGS-PCL) substrates have been extensively used as scaffolds for engineered tissues due to their desirable mechanical properties and their tunable degradability. In this study, we fabricated micro/nanofibrous scaffolds from a PGS-PCL composite using a standard electrospinning approach and then coated them with silver (Ag) using a custom radio frequency (RF) sputtering method. The Ag coating formed an electrically conductive layer around the fibers and decreased the pore size. The thickness of the Ag coating could be controlled, thereby tailoring the conductivity of the substrate. The flexible, stretchable patches formed excellent conformal contact with surrounding tissues and possessed excellent pattern-substrate fidelity. In vitro studies confirmed the platform’s biocompatibility and biodegradability. Finally, the potential controlled release of the Ag coating from the composite fibrous scaffolds could be beneficial for many clinical applications.

## 1. Introduction

Non-conventional electronics have recently attracted considerable attention in the interdisciplinary field of materials science, particularly at the interface of biology and materials [1,2]. These devices aim to combine cell-friendly materials such as polymers and electronic circuits, which could allow for low-cost, flexible, implantable, and disposable devices [3,4,5]. Such devices and systems have great promise in medical and global health applications, including environmental monitoring [6], diagnostic sensing [7], and water and food safety monitoring [8,9]. However, the fabrication of electronics on flexible polymer substrates faces many challenges due to incompatibilities with conventional integrated circuit fabrication, including chemical differences as well as the high temperatures and pressures required during inorganic microelectronics processing [10,11]. More recently, facilitated by improvements in micro- and nanotechnologies, flexible electronics have been developed using patterned organic conductors and metals on the surfaces of advanced elastomeric substrates [1,12]. These elastomeric substrates have been made from both natural (e.g., silk [13] and paper [14]) and synthetic polymer substrates (i.e., poly(dimethylsiloxane) (PDMS) [4] and poly(imide) [15]). However, novel biodegradable polymers that could be used in implantable devices have been developed more recently, including substrates made from polymers such as poly(lactic-co-glycolic) acid (PLGA) [16] and composites such as poly(glycerol sebacate) (PGS) and poly(ε-caprolactone) (PCL) [12].

**Figure 1:**
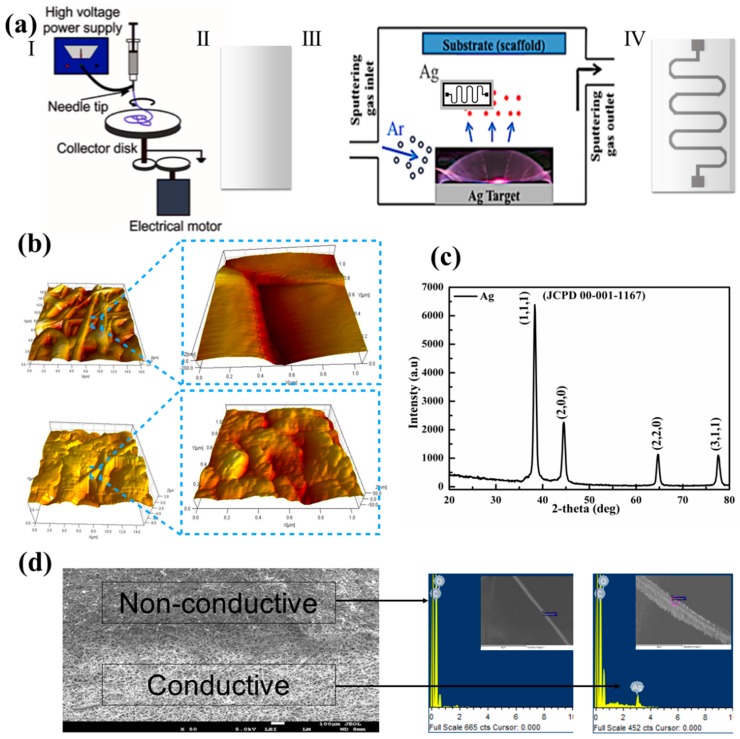
Fabrication and characterization of PCL/PGS-scaffold-substrate-based electronics. (**a**) Schematic of the electrospinning system used for fabricating sheets with uniform thickness (**i**,**ii**), schematic of the RF sputtering system used for fabricating a conductive pattern with a nano-sized silver coating (**iii**), and the tangible conductive scaffold with silver coating (**iv**). (**b**) Surface morphology of pristine and silver-coated nanofibers measured by an atomic force microscope (AFM). (**c**) X-ray diffraction (XRD) results for the silver coating on the scaffold substrate. (**d**) FESEM images of the PCL-PGS scaffold substrate showing the interface of the deposited patterns with an EDX spectra of the nanofibers (inset) on nonconductive and conductive areas showing Ag particle deposits.

The development of a new class of advanced electrical circuitry on elastomeric substrates, whose properties include flexibility, degradability, and biocompatibility, could enable the use of flexible electronics and bioactuators in novel bioengineering applications [12,17,18,19]. Flexible electronics have been fabricated by patterning metal-based microstrips on substrates that are both elastic and biodegradable [12,20,21,22]. However, to be successful, these approaches need to be based on methods that would facilitate the integration of elastic and biodegradable substrates with conductive layers that are ultimately able to maintain conformal contacts with non-flat surfaces [12,23,24]. Electrospinning is a common technique used to fabricate flexible nanofibrous substrates that can be made from various polymers (Figure 1a) [25]. To generate micro- and nanoscale electrospun fibers, a stream of a prepolymer solution is subjected to a high electrical field, and the fibers are then gathered on a collector, yielding fibrous constructs [26]. In addition to the choice of polymer, experimental electrospinning conditions, including the applied electrical field, solution flow rate and viscosity, can affect the physical properties (e.g., mechanical properties and microarchitecture) of the generated nanofibrous mats [27,28,29]. Flexible electrospun mats have previously been used for various applications, including tissue-engineered constructs and drug delivery platforms for a range of drugs. Specifically, a drug is mixed into a prepolymer solution prior to electrospinning, enabling the release of these drugs in a controlled fashion [30].

Electrospun sheets are flexible and exhibit high porosity and a microarchitecture similar to regular paper, which facilitates the fabrication of conductive patterns and flexible electronics. In addition, these sheets are easy to suture, which makes them suitable for use as implantation devices. In a recent study, electrospun fibrous substrates were used to fabricate elastic and flexible electronics. However, the elasticity of the fabricated patterns was limited due to the low pattern-substrate fidelity. In this study, we generated electrospun elastic mats from a poly(caprolactone)-poly(glycerol sebacate) (PGS-PCL) polymer mixture coated with Ag films that exhibited tunable electrical properties. These scaffolds could be used to develop constructs that are stretchable while retaining their biodegradable profiles (Figure 1). To enhance the conductive pattern’s fidelity to the substrate, electrically conductive Ag was deposited with a controlled thickness using an innovative radio frequency (RF) sputtering process. One potential application of this platform would be the development of smart wound dressings, which could provide early bacterial detection, administer drugs on demand, and reduce the required number of future visits to medical facilities.

## 2. Materials and Methods

### 2.1. Materials

Sigma-Aldrich (St. Louis, MO, USA) was the source for all lab chemicals and reagents unless mentioned otherwise. Invitrogen (Carlsbad, CA, USA) provided the biological reagents, including fetal bovine serum (FBS), Dulbecco’s modified Eagle medium (DMEM), antibiotics (penicillin/streptomycin), the LIVE/DEAD^®^ Viability/Cytotoxicity Kit and 0.05% trypsin-EDTA. Final DMEM cell culture media was prepared using 1% antibiotics (penicillin/streptomycin) supplemented with 10% FBS. To synthesize the PGS pre-polymer, we followed a previously reported protocol [31,32]. In brief, we mixed a 1:1 molar ratio of glycerol and sebacic acid at 120 °C under high vacuum to make the pre-polymer.

### 2.2. Electrospinning

The PGS and PCL (MW- 80,000 g/mol) blends were dissolved at a 1:1 ratio in a solvent mixture of chloroform and ethanol (9:1). The co-polymer concentration remained at 20% (*w*/*v*) for all constructs. To obtain a homogeneous mixture, the solutions were mixed well for 5 h at 80 °C prior to the electrospinning process. The solution was placed in a 5 mL syringe (Becton-Dickinson, Franklin Lakes, NJ, USA). A 20-gauge blunt metallic needle (NANON Supply, MECC, Fukuoka, Japan) was inserted into the syringe and attached to 10–15 cm of Teflon tubing (Cole-Parmer Instrument Company, Vernon Hills, IL, USA). An electrical field of 19.5 kV was used over a distance of 15 cm. The co-polymer solution flow rate was maintained at 0.9 mL/h. The composite was electrospun for 3 h, which yielded a thickness of 450–500 µm. The electrospun sheet was finally dried under the electrospinning system fan overnight at an airflow of 12 m^3^/h to permit solvent evaporation.

### 2.3. Fiber Coating Pattern

Silver (Ag) patterns were fabricated using an RF magnetron sputtering technique (DC/RF Magnetron Sputter System, Syskey Technologies, Xinfeng Township, Taiwan). A high purity Ag target (99.999%, 3 × 0.6 inch) was used. To generate patterns, a shadow mask prepared by a laser cutter was placed over the electrospun sheets prior to sputtering. To prepare the nanocrystalline Ag coating, a plasma was generated inside the chamber using argon gas at a flow rate of 20 sccm and an RF power of 100 watts, while the base and operating pressures were adjusted to 1 × 10^−6^ and 5 × 10^−3^ Torr, respectively. The substrate rotation, target–substrate distance, and sputtering time were 15 rpm, 14 cm and 1000 s, respectively. The thickness of the film could be controlled by increasing or decreasing the sputtering time.

### 2.4. Morphological Characterizations

Field emission scanning electron microscopy (FESEM, JEOL JSM 7600F, Tokyo, Japan) was used to determine the structural features of the fabricated conductive electrospun sheets before and after degradation assessments and mechanical testing experiments. Dried electrospun samples were mounted on copper stubs using conductive carbon adhesive tape and used for FESEM analysis. An atomic force microscope (AFM, OMICRON SPM, AFM/STM, Taunusstein, Germany) was used to determine the Ag particle size and uniformity of distributions on the micro/nanofibers after the sputtering process. X-ray diffraction (XRD) was used to confirm the crystallinity and Ag particle size.

### 2.5. Mechanical Characterization

To assess the mechanical properties of the engineered substrates, a tensile test was conducted using a mechanical tester (DMA 800 system, TA Instruments, New Castle, DE, USA) equipped with a 5 N load cell. The tensile stress (σ) was measured as a function of tensile strain (ε) at a rate of 1 N/min, in accordance with ASTM standard 882, including the testing of plastic sheets with a thickness of less than 0.25 mm.

### 2.6. Degradation and Electrical Measurements

Degradation experiments were carried out in PBS by placing the patterned electrospun mats in the buffer solution. Samples were incubated at 37 °C for 5 days. At the end of each day, the samples were removed to a dry place and dried with N_2_ before the samples were weighed. A portable multimeter with two probes was used to measure the electrical resistance of the pattern-coated sheets. The electrical resistance (*R*) of the created conductive patterns was measured over 5 days, and the ratio of the electrical resistance value after degradation (*R*) to the original electrical resistance before degradations (*R*_0_) was calculated.

### 2.7. Cell Culture

We obtained NIH 3T3 fibroblasts from American Type Culture Collection (ATCC, Manassas, VA, USA). Cell growth was carried out at 5% CO_2_ and 37 °C using DMEM media supplemented with 1% penicillin/streptomycin and 10% FBS. The passage of cells was performed in 3 day cycles, and to maintain experimental consistency, we changed the cell culture medium every 3 days.

## 3. Results and Discussion

Using an in-house-developed RF sputtering approach, we deposited different conductive patterns using silver as the RF target. A key challenge during the sputtering of metals is the increase in the substrate temperatures. In particular, if the substrate is susceptible to melting at low temperatures, it may be deformed, and the physical and architectural properties will be affected. PCL-based materials have glass and melting temperature below 60 °C and can melt during the deposition process if the substrate temperature is not controlled. In our approach, the substrate temperature was maintained below 40 °C to avoid melting. Furthermore, the deposited Ag showed excellent adhesion to the nanofibrous substrate. This excellent adhesion was mainly due to the proper coating of the nanofibers with a layer of Ag. Other patterning processes, such as printing of conductive silver inks, have resulted in poor pattern-substrate fidelity.

**Figure 2:**
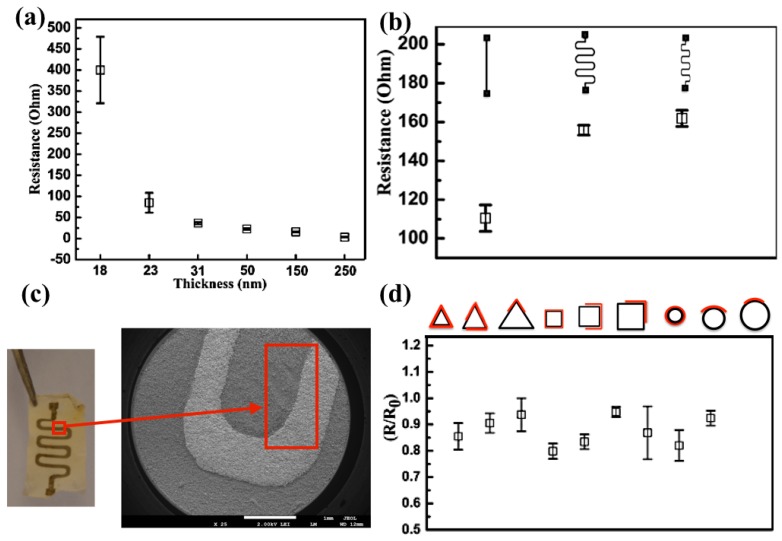
(**a**) Effect of the conductive silver pattern thickness on its electrical resistance, as measured by a multimeter. (**b**) Measurements of the electrical resistance of different patterns indicated the multiuse of the pattern structure on the surface of the electrospun substrate. (**c**) Optical image representation of the silver-coated electrospun substrate with an enlarged micrograph showing the preservation of the microstructure after the RF sputtering process. (**d**) Schematic diagram of the silver pattern on the electrospun substrate wrapped around different curved surfaces with different angles (top) and the measurements of electrical resistance after cyclic load (*R*) to the initial value of electrical resistance (*R*_0_) of the silver pattern on the substrate versus the angle to which the substrate is turned (before degradation).

Another advantage of the RF sputtering process over other methods is the ability to control the Ag layer thickness by adjusting the fabrication parameters, such as argon gas flow, gas pressure, power intensity and deposit time, which in turn enables tuning the electrical conductance (Figure 1 and Figure 2).

The patterns on the surfaces of the PGS-PCL mats could have applications in highly flexible and elastic electronics. Elastic electronics can be used for engineering strain gauges, biocompatible and elastic heaters, or temperature sensors if they are combined with other metallic materials, such as magnesium or zinc [13]. However, using Ag as the deposited pattern could also benefit other applications such as smart wound dressings, wherein the addition of thin Ag film (Figure 1b) could also impart antibacterial properties. The presence of RF-sputtered crystalline Ag film was further confirmed by analyzing the XRD pattern of the deposited film (Figure 1c) and the FESEM images in Figure 1d, in which a clear distinction between coated and uncoated fibers can be observed. Furthermore, the EDX results of the coated nanofibers in Figure 1e confirmed the presence of an Ag film. Taken together, these results suggest that the scaffolds could be applied in smart wound dressings and dermal patch applications.

RF sputtering of Ag films on the surface of PGS-PCL mats did not affect their overall microstructure because the substrate temperature was kept below 40 °C during the fabrication process (Figure 2c). Next, we analyzed the electrical and mechanical properties (i.e., conductivity and flexibility, respectively) of the Ag-patterned PGS-PCL mats with different coating thicknesses. We characterized changes in their electrical resistance/conductivity as a function of film thickness and observed that increasing the Ag film thickness enhanced the electrical conductivity of the generated patterns. The thickness could be controlled by the duration of the sputtering process and the intensity of the target evaporation. In addition, we characterized the reproducibility of our fabrication approach by analyzing the electrical properties of 3 different patterns, including a straight line (15 millimeters long and 1 millimeter wide) and two different serpentine patterns (Figure 2b). In all cases, the variation in the electrical resistance between different fabrication batches was less than 10%.

Similarly, we demonstrated the flexibility of the generated Ag film patterns by wrapping PGS-PCL mats of a single thickness (i.e., 250 nm) with a serpentine pattern around cylinders, cuboids, or triangular prisms with various diameters and heights while performing electrical resistance measurements. Depending on the positioning of the Ag pattern relative to the different prisms, the Ag film underwent different levels of tension. Figure 2d shows that the constructs remained electrically conductive over the complete range of curvature radii and different folding levels. Because the Ag film is placed on the intrinsically elastic PGS-PCL substrate, the flexibility of the conductive films can also be enhanced.

**Figure 3:**
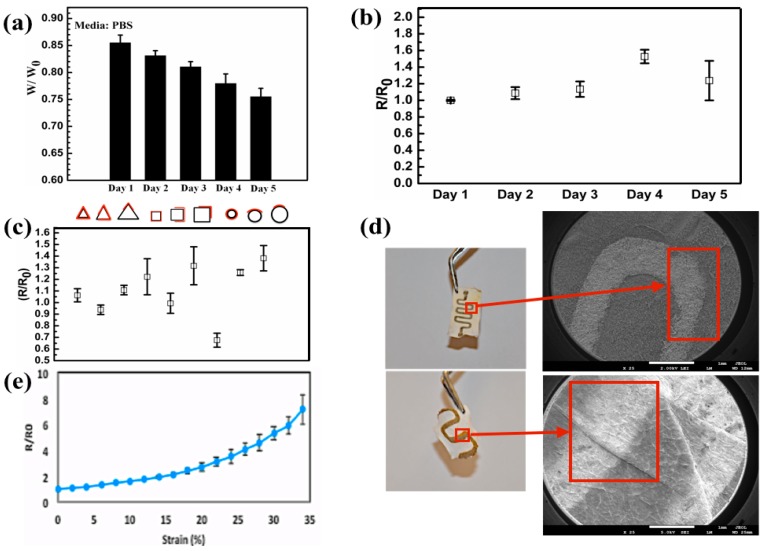
(**a**) Weight loss of the conductive patterns over 5 days in PBS. (**b**) Electrical resistance (R) of the conductive pattern over 5 days in PBS with respect to the original resistance after fabrication. (**c**) Repeated measurements from Figure 2d during degradation in PBS over 5 days. (**d**) Optical image representation of the conductive pattern after the fifth day of degradation and enlarged versions of low magnification FESEM images that show the degradation effect on the surface of the substrate. (**e**) Elasticity and flexibility of the electrospun electronics device that shows the value of electrical resistance of the stretched pattern (*R*) with the value in the un-stretched condition (*R*_0_).

To further characterize the elasticity of the fabricated patterns, we measured their electrical resistance upon stretching. We observed a less than 10% variation from each measurement in electrical conductivities for samples undergoing 20% or less strain (Figure 3), which was above the strain ranges endured by human skin [33]. Such variations in the electrical conductivity were superior to the results in previous reports using composites of silver nanoparticles on elastomeric electrospun sheets [34]. Beyond a 20% strain, these variations in electrical resistance increased, which was most likely due to the formation of cracks on the Ag film and a decrease in the contact area during the stretching of the pattern (Figure 3d). Similar observations have been made in previous studies wherein nanocomposites of elastomers incorporated with conductive particles were fabricated [35]. The relationship between electrical conductivity and tensile stress likely stems from the substrate properties. For example, as the substrate deformed, silver nanoparticles that formed a continuous conductive pattern began to separate, and the pattern cracked, which in turn affected the overall conductivity (Figure 3d). Fabricating the silver patterns through sputtering could significantly improve the overall stretching level tolerated by the patterns over previously reported methods using screen printing [36].

To test the degradation of the deposited Ag film patterns in an aqueous environment, we placed the fabricated mats in PBS. Interestingly, the electrical resistance decreased in liquid media, which might be due to water molecules penetrating into the nanosized gaps within the conductive coating. We also assessed the variation in the electrical conductivity of the sputtered patterns over a period of 5 days in PBS. We observed a maximum change of ~15% (Figure 3b) in the electrical resistance of these patterns, which perhaps occurred due to PGS-PCL physical stability under the tested conditions or detachment of electrically conductive patterns. Next, we studied the changes in electrical properties of the Ag-patterned PGS-PCL substrates as a function of their degradation. For this test, we placed nanofibrous PGS-PCL mats with serpentine Ag patterns in PBS (1×) solutions at room temperature and measured their weight and electrical resistance over a 5 day period (Figure 3a,b). Furthermore, similar to our previous analysis, we tested the flexibility of the patterns in relation to their electrical resistance after the 5 day period in PBS (Figure 3c). The changes in the electrical resistance measurements in the initial 4 days of the experiments were <10% (Figure 3b,c). However, on Day 5, we noticed a more drastic variation in the electrical resistance that was most likely due to the increased degradation of the fabricated constructs. However, we did not notice the appearance of any delamination (Figure 3d). During the degradation, the fabricated constructs exhibited an almost 25% mass loss while they were incubated in PBS (Figure 3a). These results indicate that PGS-PCL electrospun mats with Ag film pattern depositions could be used in the design of degradable electronics while retaining their electronic performance during the degradation.

**Figure 4:**
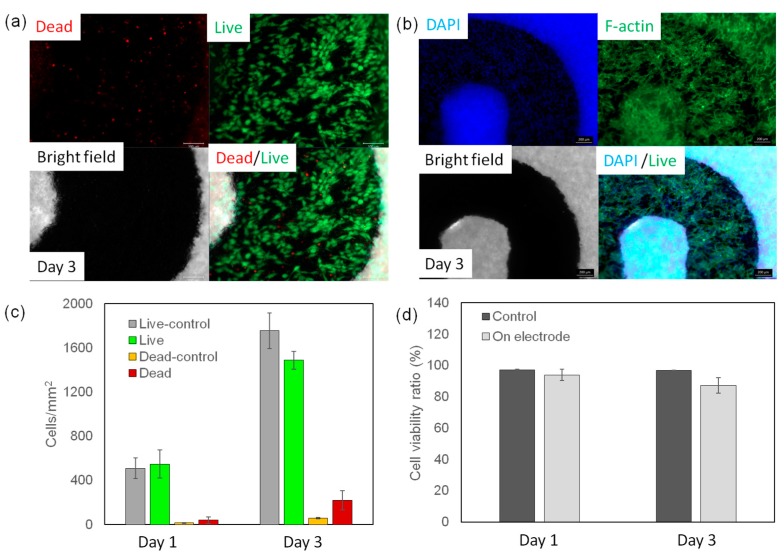
Biocompatibility of the PGS-PCL platform. To assess the biocompatibility of the platform, it was seeded with NIH 3T3 fibroblast cells with a concentration of 1 × 10^5^ per sample. (**a**) A representative fluorescence image from a cell-seeded sample and stained using a live/dead assay kit after 3 days of culture, wherein the viable cells are marked as green and dead cells are marked as red. (**b**) Rhodamine-labeled phalloidin/DAPI staining for F-actin/cell nuclei of 3T3 cells seeded on the fabricated device, which demonstrated cellular spreading on the surface of the PGS-PCL sheet after 3 days of culture. (**c,d**) The cell density and viability ratio were analyzed and were both observed to be higher than 90%.

One immediate application of such PGS-PCL substrates with integrated Ag circuitry would be engineering smart wound dressings and dermal patches that could be coupled with components for sensing and/or controlled drug release. However, since a high concentration of Ag can reduce the rate of cellular growth, silver-coated materials should be designed to prevent the rapid release of Ag or detachment of silver coatings. To assess the biocompatibility of our platform, we seeded human keratinocytes on top of the Ag-patterned PGS-PCL constructs. The constructs seeded with cells were incubated in cell culture medium at 37 °C over a period of 3 days. Cell viability on the fabricated constructs was analyzed by performing live/dead assays on Days 1 and 3 (Figure 4a,c). We observed more than 90% cell viability on both Days 1 and 3. This observation indicates that our Ag-patterned platforms did not induce significant cellular cytotoxicity over time (Figure 4c). In addition, we studied possible cellular morphological changes after 3 days of culture using staining for F-actin (Figure 4b). The staining results indicated that cells were able to spread over the entire construct surface of the PGS-PCL platform, including the area covered by the Ag film patterns. The excellent biocompatibility of the constructs was due to the slow release and detachment of silver from the nanofibrous substrate. In addition, the excellent pattern substrate fidelity achieved in the nanofibrous constructs not only improved the flexibility and reliability of the engineered circuits but also prevented rapid silver release and potential silver toxicity. Thus, the engineered substrate and fabrication process together provided an excellent tool for engineering flexible and wearable electronics. This study could be the stepping stone for further advancements in biodegradable devices, especially if the generated patterns are made from bioresorbable metals such as zinc and magnesium. These patterns could be utilized as electrical heaters to induce on-demand drug release. In addition, this study could open up new opportunities for fabricating flexible electronics and elastic devices.

## 4. Conclusions

In conclusion, we introduced polymeric nanofibrous mats fabricated with RF sputtering that were able to reproducibly deposit nanometer-scale Ag films on PGS-PCL sheets. These constructs could act as platforms for designing bioresorbable and flexible electronics in the future. In addition, this platform offers several advantages, including tunable electrical properties for a range of flexible, elastic, and degradability profiles. These PGS-PCL substrates, which were similar in structure to paper, were composed of a mesh of thin fibers that could be combined with applications commonly reserved for paper electronics. We demonstrated that our platform, which exhibited tunable electrical properties, flexibility, and elasticity of the conductive patterns, retained electrical performance when tested under environmental conditions. The electrical characteristics of the engineered platform were preserved despite gradual degradation of the PGS-PCL substrate. In addition, the PGS-PCL mats with deposited Ag films were shown to exhibit high cell compatibility during cell culturing, which indicated they would be suitable for biomedical engineering applications.
